# Fructose Malabsorption in Systemic Sclerosis

**DOI:** 10.1097/MD.0000000000001601

**Published:** 2015-10-02

**Authors:** Isabelle Marie, Anne-Marie Leroi, Guillaume Gourcerol, Hervé Levesque, Jean-François Ménard, Philippe Ducrotte

**Affiliations:** From the Department of Internal Medicine, CHU Rouen, and INSERM U 905 (IM, HL); Department of Digestive Physiology, CHU Rouen, and INSERM UMR 1073, University of Rouen IFRMP, Institute for Biochemical Research (A-ML, GG); Department of Biostatistics, CHU Rouen (J-FM); and Department of Gastroenterology, CHU Rouen, and INSERM UMR 1073, University of Rouen IFRMP, Institute for Biochemical Research, Rouen, France (PD).

## Abstract

The deleterious effect of fructose, which is increasingly incorporated in many beverages, dairy products, and processed foods, has been described; fructose malabsorption has thus been reported in up to 2.4% of healthy subjects, leading to digestive clinical symptoms (eg, pain, distension, diarrhea). Because digestive involvement is frequent in patients with systemic sclerosis (SSc), we hypothesized that fructose malabsorption could be responsible for intestinal manifestations in these patients.

The aims of this prospective study were to: determine the prevalence of fructose malabsorption, in SSc; predict which SSc patients are at risk of developing fructose malabsorption; and assess the outcome of digestive symptoms in SSc patients after initiation of standardized low-fructose diet.

Eighty consecutive patients with SSc underwent fructose breath test. All SSc patients also completed a questionnaire on digestive symptoms, and a global symptom score (GSS) was calculated.

The prevalence of fructose malabsorption was as high as 40% in SSc patients. We also observed a marked correlation between the presence of fructose malabsorption and: higher values of GSS score of digestive symptoms (*P* = 0.000004); and absence of delayed gastric emptying (*P* = 0.007). Furthermore, in SSc patients with fructose malabsorption, the median value of GSS score of digestive symptoms was lower after initiation of standardized low-fructose diet (4 before vs. 1 after; *P* = 0.0009).

Our study underscores that fructose malabsorption often occurs in SSc patients. Our findings are thus relevant for clinical practice, highlighting that fructose breath test is a helpful, noninvasive method by: demonstrating fructose intolerance in patients with SSc; and identifying the group of SSc patients with fructose intolerance who may benefit from low-fructose diet. Interestingly, because the present series also shows that low-fructose diet resulted in a marked decrease of gastrointestinal clinical manifestations in SSc patients with fructose malabsorption, our findings underscore that fructose malabsorption may play a significant role in the onset of gastrointestinal symptoms in these patients. Finally, we suggest that fructose malabsorption may be due to reduced fructose absorption by enterocytes, impaired enteric microbiome, and decreased intestinal permeability.

## INTRODUCTION

Systemic sclerosis (SSc) is a systemic inflammatory disorder affecting the skin and other organs,^1–3^ particularly the gastrointestinal tract where lesions may lead to motor activity impairment.^4–12^ Gastrointestinal involvement occurs in 50 to 88% of patients with SSc.^4–12^ Gastric abnormalities are recognized to be associated with high morbidity, resulting in gastric hemorrhage related to bleeding from antral vascular ectasia (“watermelon stomach”) and delayed gastric emptying.^4–6^ Intestinal involvement may also be responsible for severe complications, including hemorrhage due to small intestinal ectasia, malabsorption syndrome, and intestinal pseudo-obstruction.^7–12^

Recently, the deleterious effect of fermentable saccharides/polyols, especially fructose that is increasingly incorporated in many beverages, dairy products, and canned/baked or processed foods,^13–16^ has been described in non-SSc patients.^17–23^ Previous investigators have thus reported that 2.4% to 16% of healthy subjects exhibited fructose malabsorption.^21,24–27^ Other authors have further found that fructose malabsorption resulted in abdominal clinical manifestations (ie, pain, discomfort, bloating, distension, nausea, diarrhea),^21,23,26,28–30^ whereas decreased fructose intake led to significant improvement of gastrointestinal symptoms in these patients.^16,21,23,26,28–30^

Currently, however, there are no studies in SSc assessing the relationship between food intake and gastrointestinal dysfunction; in particular, the link between fructose malabsorption and the occurrence of gastrointestinal impairment has not yet been documented in SSc. Indeed, the aims of the present prospective study were to: determine the prevalence of fructose malabsorption, using fructose breath test, in unselected patients with SSc; evaluate the correlation between fructose malabsorption and digestive symptoms, nutritional status, and gastrointestinal involvement; make prediction about which SSc patients exhibit fructose malabsorption; and assess the outcome of digestive symptoms in SSc patients after initiation of standardized low-fructose diet.

## METHODS

### Patients

From January 2011 to June 2014, 80 consecutive patients with a definite diagnosis of SSc, attending a tertiary care center, were included in the study. The criteria used for the diagnosis of SSc were based on the American College of Rheumatology/European League against Rheumatism classification.^31^ Ethical approval was obtained from the local ethical committee (CERNI for the CCP de Haute Normandie), and informed consent was obtained from all patients.

The study cohort consisted of 14 men and 66 women with a median age of 52.5 years (range, 22–79 yr). The median duration of the disease, considered from the onset of the first non-Raynaud's phenomenon clinical manifestations, was 4 years (range, 1–25 yr). Patients were grouped according to criteria of LeRoy et al:^32^ 23 patients (28.8%) had diffuse cutaneous SSc (dcSSc) and 57 (71.2%) had limited cutaneous SSc (lcSSc). No patient had: other connective tissue disorders (ie, systemic lupus erythematosus, polymyositis/dermatomyositis, rheumatoid arthritis, or Sjögren's syndrome), antiphospholipid syndrome and vasculitis; and history of liver (eg, alcoholic/hepatitis B or C, cirrhosis) or digestive (eg, celiac disease, Crohn disease, ulcerative colitis) diseases, diabetes mellitus, gastric surgery, or vagotomy. Moreover, no patient received nonsteroidal anti-inflammatory drugs.

SSc patients had pulmonary involvement as follows: interstitial lung disease (ILD) (n = 25; 31.3%); and pulmonary arterial hypertension characterized by pulmonary arterial systolic pressure >40 mm Hg at rest on echocardiography (confirmed by right heart catheterization) (n = 4; 5%). Forty-three patients had digital ulcers/pitting scars (53.8%) and 31 patients had joint involvement (38.8%). Finally, 2 patients had previous history of renal crisis (2.5%).

All patients had routinely undergone esophageal manometry. Based on manometry findings, Hurwitz's criteria for degree of esophageal involvement was applied as follows: stage I: normal esophageal motility; stage II: uncoordinated peristalsis with normal pressure wave amplitude; stage III: uncoordinated peristalsis with normal low-pressure wave amplitude; and stage IV: both aperistalsis and decreased low esophageal sphincter pressure.^5–12^ According to Hurwitz's criteria, 48 patients (60%) had severe esophageal motor impairment.

Among the 80 SSc patients, 58 had routinely undergone ^13^C octanoic acid breath test, as described previously,^9^ 26 patients exhibited (44.8%) delayed gastric emptying.

### Digestive Symptoms

In our SSc patients, the median value of body mass index (BMI) was 24.2 [range, 16.8–34.5].

Before undergoing fructose breath test, SSc patients were interviewed, using a standardized questionnaire, regarding occurrence of gastrointestinal symptoms, that is, nausea, vomiting, abdominal pain/discomfort, bloating, diarrhea, constipation, abdominal tenderness, dysuria, tenesmus, fever, and general illness; each symptom carried a score from 0 (no symptoms) to 3 (severe).^9,10,33^ A global symptomatic score (GSS score), calculated as the sum of all symptom scores, was assigned to each patient (maximum score, 33), as described and validated previously.^9,10,33^ In our patients, median GSS score of digestive manifestations was 2 [range, 0–21].

Furthermore, levels of anxiety and depression were also evaluated, using the hospital anxiety and depression scales.^34^ In our patients, median values for levels of anxiety and depression were 8 [range, 2–16] and 3 [range, 0–16], respectively.

### Biochemical Tests

SSc patients routinely underwent biochemical tests. These included measurements of: first, erythrocyte sedimentation rate (ESR; mm/h), C-reactive protein (mg/L), serum albumin and prealbumin levels (g/L), ferritin (μmol/L), plasma folic acid (nmol/L), vitamin B12 (pmol/L), magnesium (mmol/L), zinc (μmol/L), selenium (μmol/L), and 25 (OH) vitamin D (nmol/L) levels; and second, antibody status: anti-centromere, anti-topoisomerase I (anti-Scl70), and anti-RNA polymerase III antibodies.

### Breath Tests

Each patient underwent 2 breath tests. First, a glucose H2/CH4 breath test was performed to exclude underlying small intestinal bacterial overgrowth (SIBO), which is a condition that may promote false-positive results of sugar breath tests.^18^ If negative, a fructose breath test was then performed after a 25 g fructose load (10% solution). If the glucose H2/CH4 breath test was positive, the patient was given antibiotic therapy, and then underwent the fructose breath test if the glucose breath test was negative.

Before both breath tests, patients were instructed to follow a diet for 48 h. Patients had been instructed to avoid foods likely to generate hydrogen for the 3 days before the test; the day preceding the examination, all patients had low carbohydrate meals (ie, nonseasoned boiled rice and meat cooked on a hot place or boiled fish and noncarbonated water). Cigarette smoking and physical exercise were not allowed for 2 h before and during the test, to avoid hyperventilation and subsequent changes in breath hydrogen.^21^ After a 12-h fasting, breath testing started after thorough mouth washing with 40 mL of 1% chlorhexidine solution to eliminate an early hydrogen peak related to action of oral bacteria on test sugars.^21^

### Glucose Breath Test

SSc patients underwent glucose H2/CH4 breath test as previously described.^10,18^ The test was considered positive for SIBO when, at least one of the following criteria was present: H_2_ and/or CH_4_ increase >20 p.p.m. above basal value; H_2_ and/or CH_4_ increase >10 p.p.m. on 2 consecutive measurements within the 2 first hours; and H_2_ and/or CH_4_ increase >10 p.p.m. between minimal and maximal values after glucose ingestion.^18,33^ When the glucose breath test was positive, patients received an antibiotic treatment with quinolone or metronidazole for 10 days per month over 3 successive months. A second glucose breath test was then performed. The fructose breath test was only made in patients exhibiting negative control glucose breath test during the next following day.

### Fructose Breath Test

Patients ingested 25 g of fructose dissolved in 250 cc of sterile water; then, end alveolar breath samples were collected every 30 min for 5 h, as described previously.^18^ Both H_2_ and CH_4_ levels were calculated. The test was considered positive and defined a fructose malabsorption in case of a rise of H_2_ and/or CH_4_ levels above 20 p.p.m.^18^

Moreover, during the test, gastrointestinal symptoms were closely monitored and were collected by the laboratory technician every 30 min of the fructose breath test. The occurrence of gastrointestinal symptoms was then analyzed and defined fructose intolerance.^18^

### Comparison of Fructose Breath Test Findings and Other Manifestations of SSc

We compared various characteristics between SSc patients with and without abnormal fructose breath test.

First, digestive manifestations were compared between these groups of SSc patients. Digestive manifestations included: the values of BMI; the values of: GSS score of digestive symptoms, anxiety and depression scales; the findings from esophageal manometry according to the Hurwitz criteria for the degree of esophageal involvement on manometry, that is, patients with and without severe esophageal motor dysfunction; and the findings from ^13^C octanoic acid breath test, that is, patients with and without delayed gastric emptying.

Second, patients with and without abnormal fructose breath test were also compared for the following characteristics: median age, median duration of SSc, distribution of SSc subsets, median modified Rodnan score, prevalence of digital ulcers/pitting scars, joint involvement, ILD, pulmonary arterial hypertension, and previous history of renal crisis; autoantibody status (anticentromere, anti-Scl70, and RNA polymerase III antibodies); and findings from biochemical tests, that is, ESR, C-reactive protein, serum albumin, and prealbumin levels, levels of ferritin, plasma folic acid, vitamin B12, magnesium/zinc/selenium, and 25 (OH) vitamin D.

### Fructose Restrictive Diet

The SSc patients with fructose intolerance were referred to an experienced dietician for a standardized diet adaptation, consisting of a 1-month low-fermentable oligo-saccharides, disaccharides, monosaccharides, and polyol (FODMAP) diet.^16^ These SSc patients were interviewed, using a standardized questionnaire regarding occurrence of gastrointestinal symptoms, using the GSS score.^5,9,10,33^

Dietary compliance was checked by direct interview by the dietician and the physician, 5 weeks after the initiation of dietary changes; compliance was considered adequate if patients confirmed that they adhered to the dietary guidelines during at least 55% of the meals consumed.

### Statistical Analysis

For group comparison involving binary data, we used either the *χ*^2^ test or Fisher exact test, depending on expected cell count (≥5 or <5, respectively). Comparisons involving continuous data were performed using: Student *t* test when distribution of variables was normal; and Mann–Whitney *U* test in other cases. The results were regarded as significant when the *P* value was less than 0.05.

## RESULTS

### Prevalence of Fructose Malabsorption

In our cohort of 80 SSc patients, the fructose breath test was positive in 32 patients (40%).

Furthermore, fructose intolerance, defined as the occurrence of gastrointestinal symptoms during fructose breath test, was found in 25 of 32 patients with SSc (78.1%).

### Predictive Factors of Fructose Malabsorption

#### Digestive Symptoms in SSc Patients

The median value for the GSS score of digestive symptoms was significantly higher in SSc patients with fructose malabsorption than in those without (4 vs. 1; *P* = 0.000004).

There were no significant differences between SSc patients with and without fructose malabsorption for: BMI levels (*P* = 0.533), scales of anxiety (*P* = 0.267), and depression (*P* = 0.148) (Table 1).

**TABLE 1:**
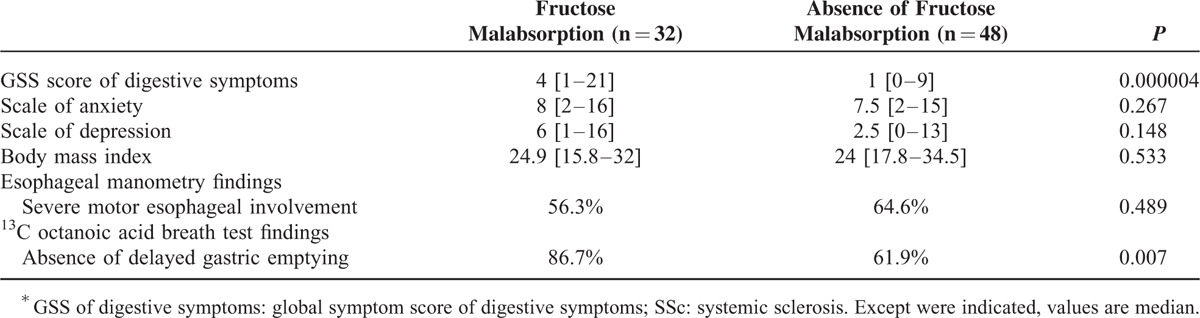
Digestive Features of SSc^∗^ Patients With Fructose Malabsorption Compared With Those Without

#### Esophageal and Gastric Involvement in SSc Patients

As shown in Table 1, patients with normal gastric emptying on ^13^C octanoic acid breath test more often exhibited fructose malabsorption (*P* = 0.007).

We failed to show any significant difference between SSc patients with and without fructose malabsorption for severe esophageal motor disorders (stage IV) on manometry (*P* = 0.489) (Table 1).

#### Other Predictive Factors for Fructose Malabsorption

##### General Clinical Characteristics

We failed to show any statistically significant differences between SSc patients with and without fructose malabsorption regarding: median age (*P* = 0.992), sex (*P* = 0.381), SSc duration (*P* = 0.487), median modified Rodnan score (*P* = 0.710); and prevalence of digital ulcers/pitting scars (*P* = 0.650), joint involvement (*P* = 0.350), ILD (*P* = 0.806), pulmonary arterial hypertension (*P* = 1) and previous history of renal crisis (*P* = 0.157) (Table 2). Additionally, fructose malabsorption tended to be more common in patients with lcSSc than in those with dcSSc, although not significantly so (*P* = 0.076). Finally, immunosuppressive therapy did not differ between SSc patients with and without fructose malabsorption for low-dose steroids (15.6% vs. 12.5%; *P* = 0.747), methotrexate (9.4% vs. 12.5%; *P* = 0.734), azathioprine (3.1% vs. 6.3%; *P* = 0.646), and mycophenolate mofetil (15.6% vs. 6.3%; *P* = 0.256).

**TABLE 2:**
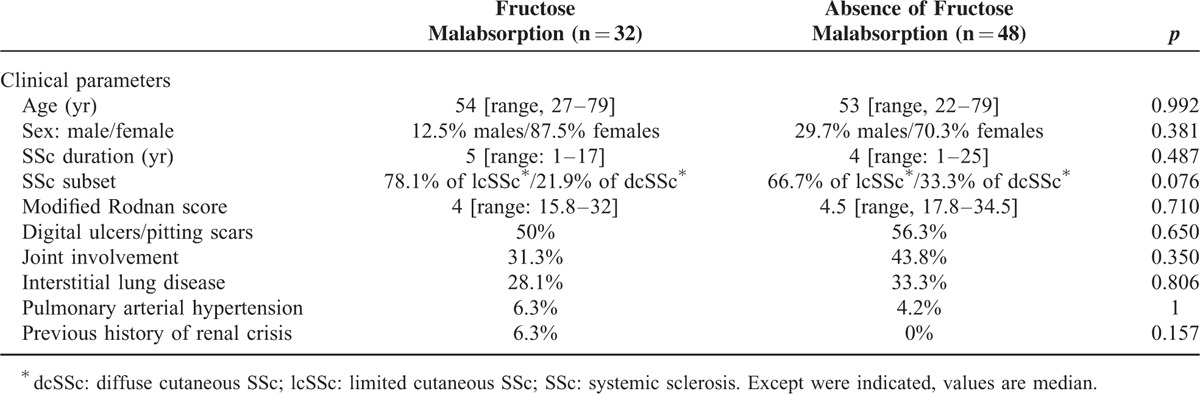
Clinical Characteristics of SSc^∗^ Patients With Fructose Malabsorption Compared With Those Without

##### Laboratory Findings

As seen in Table 3, regarding biochemical tests, SSc patients with and without fructose malabsorption did not exhibit different median levels of ESR (*P* = 0.579), C-reactive protein (*P* = 0.827), albumin (*P* = 0.276), prealbumin (*P* = 0.455), ferritin (*P* = 0.941), plasma folic acid (*P* = 0.444), vitamin B12 (*P* = 0.115), magnesium (*P* = 0.835), zinc (*P* = 0.677), selenium (*P* = 0.077), and 25 (OH) vitamin D (*P* = 0.155).

**TABLE 3:**
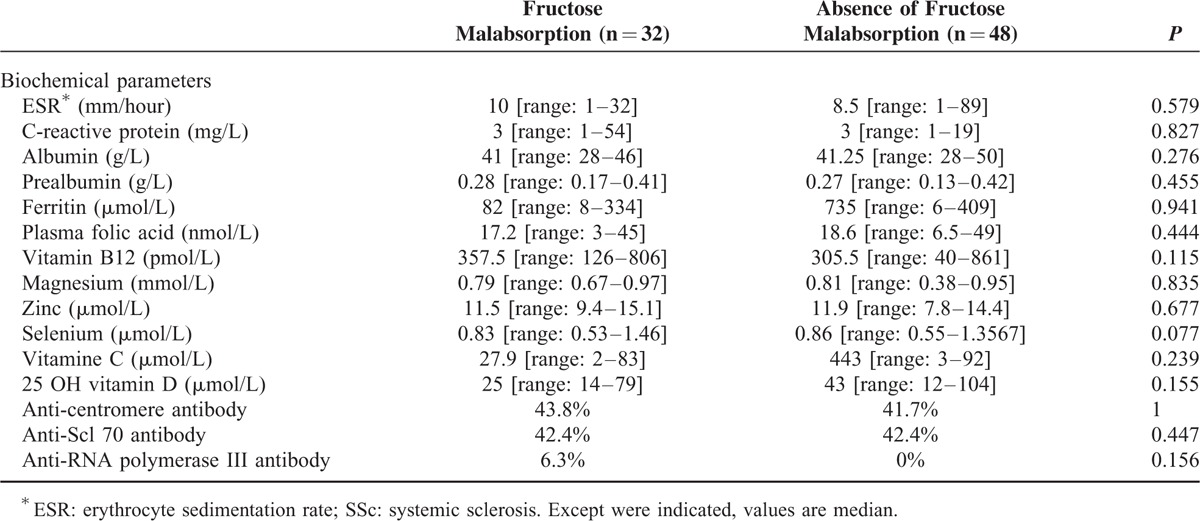
Biochemical Characteristics of SSc^∗^ Patients With Fructose Malabsorption Compared With Those Without

Additionally, autoantibody status did not differ between SSc patients and without fructose malabsorption for anti-centromere (*P* = 1), anti-Scl 70 (*P* = 0.447), and anti-RNA polymerase III (*P* = 0.156) antibodies (Table 3).

#### Outcome of Gastrointestinal Symptoms After Low-FODMAP Diet

Complete outcomes were available in all 32 SSc patients with fructose malabsorption who received low-FODMAP diet. Furthermore, 91% of these patients reported adequate dietary compliance. In overall 32 SSc patients, who received low-FODMAP diet, other specific therapy of SSc was unchanged.

As shown in Table 4, after low-FODMAP diet initiation, SSc patients with fructose malabsorption less commonly exhibited: nausea (*P* = 7.09 × 10^−6^), vomiting (*P* = 0.024), abdominal pain (*P* = 6.62 × 10^−9^), bloating (*P* = 0.0009), diarrhea (*P* = 0.022), and abdominal tenderness (*P* = 0.008). In addition, median value of GSS score of digestive symptoms was significantly lower (4 before vs. 1 after; *P* = 0.00009) after initiation of low-FODMAP diet in SSc patients.

**TABLE 4:**
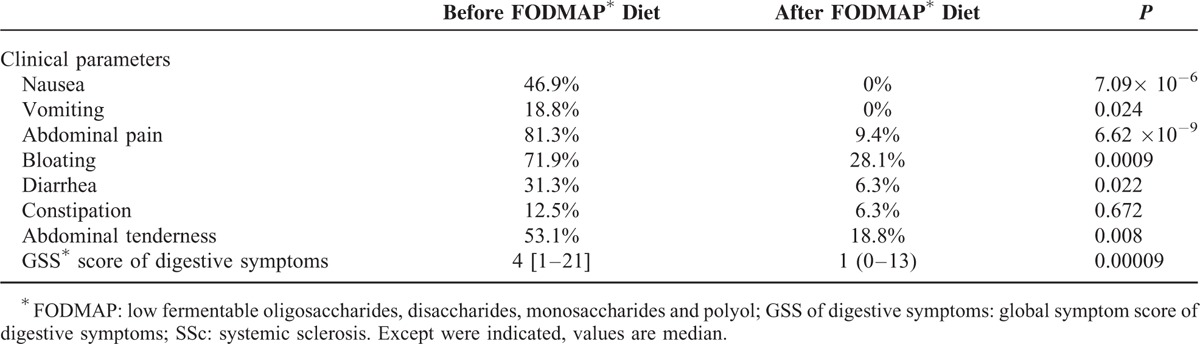
Gastrointestinal Features of the 32 SSc^∗^ Patients With Fructose Malabsorption Before and After FODMAP^∗^ Diet Initiation

## DISCUSSION

Fructose is a 6-carbon monosaccharide molecule that is found in fruits and vegetables; furthermore, it is increasingly incorporated in processed foods.^13–16,18,23^ Fructose malabsorption has been shown to be responsible for gastrointestinal symptoms.^16–23,26,28–30^ In clinical practice, fructose breath test has been reported as a helpful and reliable method for assessment of fructose malabsorption.^17,18,20,21,35,36^ However, to date, there have been no large studies using fructose breath test in SSc and consequently, the prevalence of fructose malabsorption has not yet been determined in these patients.

The present prospective study is, to the best of our knowledge, the first to evaluate fructose malabsorption in SSc. In this instance, we found a high frequency (40%) of fructose malabsorption in patients with SSc. Since we investigated 80 SSc patients without any prior selection based on clinical and digestive presentation, our sample appeared to be representative of the entire SSc population. Our findings therefore underscore that fructose malabsorption is prevalent in the whole population of SSc patients. In the current study, fructose breath test was not performed in healthy controls, since previous investigators reported that these latter subjects exhibited fructose malabsorption in only 2.4 to 16% of cases.^21,24–27^ Other authors have also observed fructose malabsorption, in 14.4 to 39% of patients with irritable bowel syndrome (IBS).^21,37,38^

The current study interestingly underscores that fructose breath test is a reliable method for noninvasively disclosing fructose malabsorption in SSc. Our experimental protocol was carefully designed to avoid any methodological bias that could interfere with the interpretation of the test.

First, SIBO can reasonably be ruled out as a cause of increased proportion of fructose malabsorption in our SSc patients; indeed, all these patients had a negative glucose breath test making SIBO very unlikely. Indeed, because colonic bacteria population migrates proximally into the small intestine in SIBO (gaining access to sugars), this shift in fermentation has been reported to lead to abnormal fructose breath test.^39,40^ However, 1 potential limitation of our study was the use of a glucose breath test for SIBO diagnosis. In fact, the gold standard for the diagnosis of SIBO remains the culture of small bowel aspirates, even such aspirates cannot detect bacterial overgrowth in the distal jejunum and proximal ileum.

Second, we chose 25 g as the dose of fructose load for testing SSc patients, because it is usually accepted as the appropriate dose for clinical use of fructose breath testing.^21,25,27,41–47^ Although Goebel-Stengel et al^48,49^ used 50 g as the dose of fructose load, other authors have shown that healthy subjects can only absorb up to 25 g of fructose per day; indeed, while it is possible that some healthy volunteers may consume up to 50 g of fructose per day, this amount is usually not ingested all at once.^21,25,27,41–47,50,51^ In a series of 17 healthy controls, increasing fructose consumption from 15–25 g to 50 g resulted in increased prevalence of fructose malabsorption from 5.9% to 52.9%.^43^ Another series, which compared 3 doses of fructose load (15, 25, and 50 g), also demonstrated that 100% of healthy volunteers could absorb 15 g of fructose, 90% could absorb 25 g of fructose, but only 20 to 30% could absorb 50 g.^25^ Altogether these findings suggest that the physiologic dose of 25 g fructose appears to be relevant to characterize clinically significant fructose malabsorption, higher doses leading to false-positive diagnosis of fructose malabsorption.^42^

Furthermore, we used a 5-h fructose breath test duration, because previous authors have found that 10 to 16% of fructose malabsorption would be missed with a 3-h fructose breath test duration.^18,35^ In this instance, the collection of breath samples was also performed according to previous methodological recommendations to ensure end-alveolar breath samples;^52^ we measured both H2 and CH4 levels, as it has been shown that false-negative findings were observed in 14% of cases when CH4 is not measured.^53,54^^.^

Another main finding in the present series was that fructose malabsorption was strongly associated with a greater prevalence and intensity of gastrointestinal clinical manifestations in SSc, as shown by the higher median value of the GSS score of digestive symptoms in SSc patients exhibiting fructose malabsorption. Previous investigators have indeed reported that unabsorbed fructose will reach the colon in patients, where colonic fermentation leads to gas production (including hydrogen, carbon dioxide, hydrogen sulphide, methan, and short-chain fatty acids), resulting in gastrointestinal symptoms such as abdominal pain and discomfort, bloating, distension, nausea, and diarrhea.^18,21,23,29,48,54–57^ In our experience, SSc patients with fructose malabsorption did not show a more altered psychological profile than SSc patients without fructose malabsorption, as shown by similar scales of anxiety and depression.

Taken together, our data suggest that fructose breath test should be performed in SSc patients without SIBO exhibiting unexplained clinical gastrointestinal symptoms to detect underlying fructose malabsorption.

In the current study, neither the patients’ age nor SSc duration could be considered to be predictive of fructose malabsorption. Furthermore, our study shows that the prevalence of fructose malabsorption was similar in patients with dcSSc and lcSSc. In addition, we found that fructose malabsorption was not correlated with other extradigestive manifestations of SSc, including digital ulcers/pitting scars, joint involvement, ILD, pulmonary arterial hypertension, and previous history of renal crisis. Finally, the present study also shows that autoantibody status, including anticentromere, anti-Scl 70 nd anti-RNA polymerase III antibodies, could not be considered predictive factors of fructose malabsorption in patients with SSc.

Additionally, our findings raise the question of whether fructose malabsorption is a participating mechanism or an epiphenomenon in gastrointestinal involvement related to SSc.

From a physiologic standpoint, the human small intestine lacks enzyme to digest fructose; fructose is slowly absorbed across the intestinal epithelium by carrier-mediated facilitated diffusion, which is an energy-independent process.^35,58^ The fructose carrier is a member of the glucose transport (GLUT) family of genes encoding for facilitative sugar transporters.^58^ Previous authors have shown that fructose is mainly absorbed through: GLUT5, which is found in the apical membrane on the luminal surface of human small intestine enterocytes;^52^ and GLUT2 (which is located in the basolateral membrane of intestinal epithelial cells), becoming relevant when high doses of fructose are ingested.^35,58–61^ Other fructose transporters have been described, including GLUT7, GLUT8, and GLUT12, allowing adaptation to high free fructose intake.^62–64^ In a GLUT5 knockout murine model, fructose absorption was decreased by 75% in the jejunum compared with wild-type mice;^65^ these knockout mice, fed with high-fructose diet, developed intestinal dilation and fluid retention reminiscent of fructose malabsorption in humans.^66^ Interestingly, higher prevalence of fructose malabsorption has been reported in patients exhibiting decreased expression of GLUT5;^67,68^ furthermore, in patients with noninsulin-dependent diabetes mellitus, increased expression of GLUT5 has been shown to be correlated with increased absorption of fructose.^69^ By contrast, fructose malabsorption is not associated with GLUT5/GLUT2 mutation.^66^ Recently, Wilder-Smith et al^35^ have suggested that expression of the GLUT5/GLUT2 transporters may not be uniform along the small intestine. In SSc patients, we suggest that fructose malabsorption may also be, in part, explained by reduced fructose absorption by the enterocytes related to decreased expression of GLUT5/GLUT2 transporters. In essence, inflammation has been shown to decrease intestinal GLUT5 activity and mRNA expression in murine models;^63^ similarly, in SSc patients, histological analyses of biopsy specimens have shown mononuclear inflammatory cells within intestinal wall. However, a limitation of the current study is that we did not assess the expression of GLUT5/GLUT2 transporter proteins in our SSc patients; thus, other investigations are warranted to confirm these data.

Another main finding in the present series is that fructose malabsorption was more frequent in our SSc patients with normal gastric emptying. Indeed, in healthy subjects, faster is gastric emptying, higher is the fructose load in the intestine and subsequently, higher is the osmotic load related to fructose, leading to fructose intolerance; it has been postulated that in patients with delayed gastric emptying, the postprandial small intestinal load of fructose is lower, reducing the osmotic effect of fructose, reducing the osmotic effect of fructose subsequently.^70–72^ Our findings are in accordance with this hypothesis, as SSc patients with normal gastric emptying more often exhibited fructose malabsorption.

Additionally, in SSc patients, we also suggest that fructose malabsorption may be related to: collagenous fibrosis of small intestinal wall, resulting in decreased intestinal permeability and fructose malabsorption, subsequently;^4^ and impairment of enteric microbiome. Previous investigators have reported that fructose increases intestinal translocation of bacterial endotoxin and subsequent activation of Kupffer cells through TLR-dependent mechanisms in mice.^72,73^ However, no definite conclusion can be drawn from our findings, and further investigations are warranted to confirm these data.

Another finding in the present study was that SSc patients with fructose malabsorption more often exhibited fructose intolerance (78.1% of cases), determined by the onset of gastrointestinal clinical manifestations during the fructose breath test. We thus suggest that fructose breath test may be useful in identifying the group of SSc patients with fructose intolerance who may benefit from low-fructose diet.

In fact, fructose coexists in food with other poorly absorbed carbohydrates that are FODMAPS.^19^ The low-FODMAD diet specifically limits the intake of poorly absorbed short-chain carbohydrates, inducing luminal distension by promoting osmosis and colonic gas production.^23^ Previous authors have reported that the low-FODMAP diet is helpful in: IBS patients with symptomatic fructose intolerance, resulting in rapid improvement (within 1 wk) of gastrointestinal manifestations;^14,19,23,29,73–77^ and patients with nonceliac gluten sensitivity, leading to improvement of gastrointestinal symptoms.^78^ The hypothetized mechanism of action of low-FODMAP diet is related to decreased microbial fermentation of dietary FODMAPs, resulting in lower luminal gas generation and distension.^79^ Until now, no studies have directly compared the efficacy of restricting all FODMAPS with that of restricting fructose/fructans alone in patients exhibiting fructose malabsorption. In 1 study, low-fructose/fructan diet was found to reduce digestive symptoms in patients with fructose malabsorption.^80^ Altogether, to date, low-FODMAP diet has been mainly recommended in patients with symptomatic fructose malabsorption.^81^

The present series also suggests that low-FODMAP diet is useful in SSc patients with symptomatic fructose malabsorption, resulting in marked improvement of clinical manifestations (ie, nausea, vomiting, abdominal pain, bloating, diarrhea, and abdominal tenderness). Our findings thus underscore that fructose malabsorption may play a significant role in the onset of gastrointestinal symptoms in SSc patients without SIBO. Interestingly, we have also observed that the strategy of dietary advice, being delivered by trained dietician following fructose breath test, provided an appropriate base for SSc patients to adhere to low-FODMAP diet which is crucial for its success. Although there are limitations in our study, because this is a nonrandomized controlled study with the potential for a placebo effect from the intervention; further randomized series are therefore warranted to confirm our findings.

In conclusion, we report a high prevalence (40%) of fructose malabsorption in SSc. Our findings are thus relevant for clinical practice, highlighting that fructose breath test is a helpful, noninvasive method by: demonstrating fructose intolerance in patients with SSc; and identifying the group of SSc patients with fructose intolerance who may benefit from fructose restriction diet. Interestingly, because the present series also shows that low-FODMAP diet resulted in a marked decrease of gastrointestinal clinical manifestations in SSc patients with fructose malabsorption, our findings underscore that fructose malabsorption may play a significant role in the onset of gastrointestinal symptoms in these patients. Finally, we suggest that fructose malabsorption may be related to impairment of fructose absorption by the enterocytes and/or enteric microbiome and decrease of intestinal permeability in SSc.

## References

[1] Marie I Gehanno JF Environmental risk factors of systemic sclerosis. *Semin Immunopathol* 2015; 37:463–473. 26141606 10.1007/s00281-015-0507-3

[2] Marie I Gehanno JF Bubenheim M Prospective study to evaluate the association between systemic sclerosis and occupational exposure and review of the literature. *Autoimmun Rev* 2014; 13:151–156. 24129037 10.1016/j.autrev.2013.10.002

[3] Marie I Menard JF Duval-Modeste AB Association of occupational exposure with features of systemic sclerosis. *J Am Acad Dermatol* 2015; 72:456–464. 25582539 10.1016/j.jaad.2014.11.027

[4] Forbes A Marie I Gastrointestinal complications: the most frequent internal complications of systemic sclerosis. *Rheumatology (Oxford)* 2009; 48:iii36–iii39. 19487222 10.1093/rheumatology/ken485

[5] Marie I Antonietti M Houivet E Gastrointestinal mucosal abnormalities using videocapsule endoscopy in systemic sclerosis. *Aliment Pharmacol Ther* 2014; 40:189–199. 24889779 10.1111/apt.12818

[6] Marie I Ducrotte P Antonietti M Watermelon stomach in systemic sclerosis: its incidence and management. *Aliment Pharmacol Ther* 2008; 28:412–421. 18498445 10.1111/j.1365-2036.2008.03739.x

[7] Marie I Dominique S Levesque H Esophageal involvement and pulmonary manifestations in systemic sclerosis. *Arthritis Rheum* 2001; 45:346–354. 11501722 10.1002/1529-0131(200108)45:4<346::AID-ART347>3.0.CO;2-L

[8] Marie I Ducrotté P Denis P Oesophageal mucosal involvement in patients with systemic sclerosis receiving proton pump inhibitor therapy. *Aliment Pharmacol Ther* 2006; 24:1593–1601. 17206947 10.1111/j.1365-2036.2006.03180.x

[9] Marie I Ducrotté P Denis P Outcome of small bowel motor impairment in systemic sclerosis: a prospective manometric 5-year follow-up. *Rheumatology (Oxford)* 2007; 46:150–153. 16782730 10.1093/rheumatology/kel203

[10] Marie I Gourcerol G Leroi AM Delayed gastric emptying determined using the 13C-octanoic acid breath test in patients with systemic sclerosis. *Arthritis Rheum* 2012; 64:2346–2355. 22231388 10.1002/art.34374

[11] Marie I Leroi AM Menard JF Fecal calprotectin in systemic sclerosis and review of the literature. *Autoimmun Rev* 2015; 14:547–554. 25661980 10.1016/j.autrev.2015.01.018

[12] Marie I Levesque H Ducrotté P Gastric involvement in systemic sclerosis: a prospective study. *Am J Gastroenterol* 2001; 41:1874–1883. 10.1111/j.1572-0241.2001.03353.x 11197291

[13] Bray GA Nielsen SJ Popkin BM Consumption of high-fructose corn syrup in beverages may play a role in the epidemic of obesity. *Am J Clin Nutr* 2004; 79:537–543. 15051594 10.1093/ajcn/79.4.537

[14] Choi YK Kraft N Zimmerman B Fructose intolerance in IBS and utility of fructose-restricted diet. *J Clin Gastroenterol* 2008; 42:233–238. 18223504 10.1097/MCG.0b013e31802cbc2f

[15] Economic Research Service, USDA. Table 49-US total estimated deliveries of caloric sweeteners for domestic food and beverage use, by calendar year 2003.

[16] Fedewa A Rao SS Dietary fructose intolerance, fructan intolerance and FODMAPs. *Curr Gastroenterol Rep* 2014; 16:370. 24357350 10.1007/s11894-013-0370-0 PMC3934501

[17] Riby JE Fujisawa T Kretchmer N Fructose absorption. *Am J Clin Nutr* 1993; 58:748S–753S. 8213606 10.1093/ajcn/58.5.748S

[18] Melchior C Gourcerol G Déchelotte P Symptomatic fructose malabsorption in irritable bowel syndrome: a prospective study. *United European Gastroenterol J* 2014; 2:131–137. 10.1177/2050640614521124 PMC4040818 24918018

[19] Putkonen L Yao CK Gibson PR Fructose malabsorption syndrome. *Curr Opin Clin Nutr Metab Care* 2013; 16:473–477. 23739630 10.1097/MCO.0b013e328361c556

[20] Ragnarsson G Bodemar G Pain is temporally related to eating but not to defaecation in the irritable bowel syndrome (IBS). Patients’ description of diarrhea, constipation and symptom variation during a prospective 6-week study. *Eur J Gastroenterol Hepatol* 1998; 10:415–421. 9619389 10.1097/00042737-199805000-00011

[21] Sharma A Srivastava D Verma A Fructose malabsorption is not uncommon among patients with irritable bowel syndrome in India: a case-control study. *Indian J Gastroenterol* 2014; 33:466–470. 25066182 10.1007/s12664-014-0492-9

[22] Shepherd SJ Parker FC Muir JG Dietary triggers of abdominal symptoms in patients with irritable bowel syndrome: randomized placebo-controlled evidence. *Clin Gastroenterol Hepatol* 2008; 6:765–771. 18456565 10.1016/j.cgh.2008.02.058

[23] Turnbull JL Adams HN Gorard DA The diagnosis and management of food allergy and food intolerances. *Aliment Pharmacol Ther* 2015; 41:3–25. 25316115 10.1111/apt.12984

[24] Densupsoontorn N Jirapinyo P Thamonsiri N Fructose malabsorption in Thai adult. *Asia Pac J Clin Nutr* 2007; 16:209–212. 17468074

[25] Rao SS Attaluri A Anderson L Ability of the normal human small intestine to absorb fructose: evaluation by breath testing. *Clin Gastroenterol Hepatol* 2007; 5:959–963. 17625977 10.1016/j.cgh.2007.04.008 PMC1994910

[26] Reyes-Huerta JU de la Cruz-Patiño E Ramírez-Gutiérrez de Velasco A Fructose intolerance in patients with irritable bowel syndrome: a case-control study. *Rev Gastroenterol Mex* 2010; 75:405–411. 21169107

[27] Truswell AS Seach JM Thorburn AW Incomplete absorption of pure fructose in healthy subjects and the facilitating effect of glucose. *Am J Clin Nutr* 1988; 48:1424–1430. 3202090 10.1093/ajcn/48.6.1424

[28] de Roest RH Dobbs BR Chapman BA The low FODMAP diet improves gastrointestinal symptoms in patients with irritable bowel syndrome: a prospective study. *Int J Clin Pract* 2013; 67:895–903. 23701141 10.1111/ijcp.12128

[29] Halmos EP Power VA Shepherd SJ A diet low in FODMAPs reduces symptoms of irritable bowel syndrome. *Gastroenterology* 2014; 146:67–75. 24076059 10.1053/j.gastro.2013.09.046

[30] Staudacher HM Whelan K Irving PM Comparison of symptom response following advice for a diet low in fermentable carbohydrates (FODMAPs) versus standard dietary advice in patients with irritable bowel syndrome. *J Hum Nutr Diet* 2011; 24:487–495. 21615553 10.1111/j.1365-277X.2011.01162.x

[31] van den Hoogen F Khanna D Fransen J 2013 classification criteria for systemic sclerosis: an American College of Rheumatology/European League against Rheumatism collaborative initiative. *Arthritis Rheum* 2013; 65:2737–2747. 24122180 10.1002/art.38098 PMC3930146

[32] Leroy EC Black C Fleischmajer R Scleroderma (systemic sclerosis): classification, subsets and pathogenesis. *J Rheumatol* 1988; 15:202–205. 3361530

[33] Marie I Ducrotté P Denis P Small intestinal bacterial overgrowth in systemic sclerosis. *Rheumatology (Oxford)* 2009; 48:1314–1319. 19696066 10.1093/rheumatology/kep226

[34] Cho HS Park JM Lim CH Anxiety, depression and quality of life in patients with irritable bowel syndrome. *Gut Liver* 2011; 5:29–36. 21461069 10.5009/gnl.2011.5.1.29 PMC3065090

[35] Wilder-Smith CH Materna A Wermelinger C Fructose and lactose intolerance and malabsorption testing: the relationship with symptoms in functional gastrointestinal disorders. *Aliment Pharmacol Ther* 2013; 37:1074–1083. 23574302 10.1111/apt.12306 PMC3672687

[36] Romagnuolo J Schiller D Bailey RJ Using breath tests wisely in a gastroenterology practice: an evidence-based review of indications and pitfalls in interpretation. *Am J Gastroenterol* 2002; 97:1113–1126. 12014715 10.1111/j.1572-0241.2002.05664.x

[37] Choi YK Johlin FC Jr Summers RW Fructose intolerance: an under-recognized problem. *Am J Gastroenterol* 2003; 98:1348–1353. 12818280 10.1111/j.1572-0241.2003.07476.x

[38] Corlew-Roath M Di Palma JA Clinical impact of identifying lactose maldigestion or fructose malabsorption in irritable bowel syndrome or other conditions. *South Med J* 2009; 102:1010–1012. 19738525 10.1097/SMJ.0b013e3181b64c7f

[39] Montalto M Gallo A Ojetti V Fructose, trehalose and sorbitol malabsorption. *Eur Rev Med Pharmacol Sci* 2013; 17 Suppl 2:26–29. 24443064

[40] Posserud I Stotzer PO Björnsson ES Small intestinal bacterial overgrowth in patients with irritable bowel syndrome. *Gut* 2007; 56:802–808. 17148502 10.1136/gut.2006.108712 PMC1954873

[41] Berg LK Fagerli E Martinussen M Effect of fructose-reduced diet in patients with irritable bowel syndrome, and its correlation to a standard fructose breath test. *Scand J Gastroenterol* 2013; 48:936–943. 23834159 10.3109/00365521.2013.812139

[42] Erdogan A Adame EC Yu S Optimal testing for diagnosis of fructose intolerance: over-dosage leads to false positive intolerance test. *J Neurogastroenterol Motil* 2014; 20:560. 25273127 10.5056/jnm14085 PMC4204421

[43] Frieling T Kuhlbusch-Zicklam R Kalde S Fructose malabsorption: how much fructose can a healthy subject tolerate? *Digestion* 2011; 84:269–272. 21952629 10.1159/000329570

[44] Gibson PR Newnham E Barrett JS Review article: fructose malabsorption and the bigger picture. *Aliment Pharmacol Ther* 2007; 25:349–363. 17217453 10.1111/j.1365-2036.2006.03186.x

[45] Ravich WJ Bayless TM Thomas M Fructose: incomplete intestinal absorption in humans. *Gastroenterology* 1983; 84:26–29. 6847852

[46] Rumessen JJ Gudmand-Høyer E Absorption capacity of fructose in healthy adults. Comparison with sucrose and its constituent monosaccharides. *Gut* 1986; 27:1161–1168. 3781328 10.1136/gut.27.10.1161 PMC1433856

[47] Sung HY Kim YS Fructose malabsorption in patients with irritable bowel syndrome-like symptoms: What is the role in the pathogenesis and clinical implication? *J Neurogastroenterol Motil* 2014; 20:135–137. 24840365 10.5056/jnm.2014.20.2.135 PMC4015205

[48] Goebel-Stengel M Stengel A Schmidtmann M Unclear abdominal discomfort: pivotal role of carbohydrate malabsorption. *J Neurogastroenterol Motil* 2014; 20:228–235. 24840375 10.5056/jnm.2014.20.2.228 PMC4015196

[49] Goebel-Stengel M Monnikes H Optimal testing for diagnosis of fructose malabsorption: under-dosage leads to false negative intolerance test. *J Neurogastroenterol Motil* 2015; 21:296–297. 25843084 10.5056/jnm14153 PMC4398236

[50] Kyaw MH Mayberry JF Fructose malabsorption: true condition or a variance from normality. *J Clin Gastroenterol* 2011; 45:16–21. 20818234 10.1097/MCG.0b013e3181eed6bf

[51] Montonen J Järvinen R Knekt P Consumption of sweetened beverages and intakes of fructose and glucose predict type 2 diabetes occurrence. *J Nutr* 2007; 137:1447–1454. 17513405 10.1093/jn/137.6.1447

[52] Pimentel M Chow EJ Lin HC Eradication of small intestinal bacterial overgrowth reduces symptoms of irritable bowel syndrome. *Am J Gastroenterol* 2000; 95:3503–3506. 11151884 10.1111/j.1572-0241.2000.03368.x

[53] Knudsen CD Di Palma JA Do you need to measure methane? *South Med J* 2012; 105:251–253. 22561536 10.1097/SMJ.0b013e318252d428

[54] de Lacy Costello BP Ledochowski M Ratcliffe NM The importance of methane breath testing: a review. *J Breath Res* 2013; 7: 024001.38. 10.1088/1752-7155/7/2/024001 23470880

[55] Latulippe ME Skoog SM Fructose malabsorption and intolerance: effects of fructose with and without simultaneous glucose ingestion. *Crit Rev Food Sci Nutr* 2011; 51:583–592. 21793722 10.1080/10408398.2011.566646 PMC3471321

[56] Ong DK Mitchell SB Barrett JS Manipulation of dietary short chain carbohydrates alters the pattern of gas production and genesis of symptoms in irritable bowel syndrome. *J Gastroenterol Hepatol* 2010; 25:1366–1373. 20659225 10.1111/j.1440-1746.2010.06370.x

[57] Simrén M Stotzer PO Use and abuse of hydrogen breath tests. *Gut* 2006; 55:297–303. 16474100 10.1136/gut.2005.075127 PMC1856094

[58] Biesiekierski JR Fructose-induced symptoms beyond malabsorption in FGID. *United European Gastroenterol J* 2014; 2:10–13. 10.1177/2050640613510905 PMC4040804 24918003

[59] Burant CF Takeda J Brot-Laroche E Fructose transporter in human spermatozoa and small intestine is GLUT5. *J Biol Chem* 1992; 267:14523–14526. 1634504

[60] Davidson NO Hausman AM Ifkovits CA Human intestinal glucose transporter expression and localization of GLUT5. *Am J Physiol* 1992; 262:C795–C800. 1550217 10.1152/ajpcell.1992.262.3.C795

[61] Gouyon F Onesto C Dalet V Fructose modulates GLUT5 mRNA stability in differentiated Caco-2 cells: role of cAMP-signalling pathway and PABP (polyadenylated-binding protein)-interacting protein (Paip) 2. *Biochem J* 2003; 375:167–174. 12820898 10.1042/BJ20030661 PMC1223656

[62] DeBosch BJ Chi M Moley KH Glucose transporter 8 (GLUT8) regulates enterocyte fructose transport and global mammalian fructose utilization. *Endocrinology* 2012; 153:4181–4191. 22822162 10.1210/en.2012-1541 PMC3423610

[63] Douard V Ferraris RP Regulation of the fructose transporter GLUT5 in health and disease. *Am J Physiol Endocrinol Metab* 2008; 295:E227–237. 18398011 10.1152/ajpendo.90245.2008 PMC2652499

[64] Jones HF Butler RN Brooks DA Intestinal fructose transport and malabsorption in humans. *Am J Physiol Gastrointest Liver Physiol* 2011; 300:G202–G206. 21148401 10.1152/ajpgi.00457.2010

[65] Vos MB Nutrition, nonalcoholic fatty liver disease and the microbiome: recent progress in the field. *Curr Opin Lipidol* 2014; 25:61–66. 24366230 10.1097/MOL.0000000000000043 PMC3947892

[66] Wasserman D Hoekstra JH Tolia V Molecular analysis of the fructose transporter gene (GLUT5) in isolated fructose malabsorption. *J Clin Invest* 1996; 98:2398–2402. 8941659 10.1172/JCI119053 PMC507692

[67] Douard V Ferraris RP The role of fructose transporters in diseases linked to excessive fructose intake. *J Physiol* 2013; 591:401–414. 23129794 10.1113/jphysiol.2011.215731 PMC3577529

[68] Jones HF Butler RN Moore DJ Developmental changes and fructose absorption in children: effect on malabsorption testing and dietary management. *Nutr Rev* 2013; 71:300–309. 23590706 10.1111/nure.12020

[69] Dyer J Wood IS Palejwala A Expression of monosaccharide transporters in intestine of diabetic humans. *Am J Physiol Gastrointest Liver Physiol* 2002; 282:G241–G248. 11804845 10.1152/ajpgi.00310.2001

[70] Southgate DA Digestion and metabolism of sugars. *Am J Clin Nutr* 1995; 62:203S–210S. 7598078 10.1093/ajcn/62.1.203S

[71] Horowitz M Cunningham KM Wishart JM The effect of short-term dietary supplementation with glucose on gastric emptying of glucose and fructose and oral glucose tolerance in normal subjects. *Diabetologia* 1996; 39:481–486. 8777999 10.1007/BF00400681

[72] Moukarzel AA Sabri MT Gastric physiology and function: effects of fruit juices. *J Am Coll Nutr* 1996; 15 (5 Suppl):18S–25S. 8892179 10.1080/07315724.1996.10720470

[73] Barrett JS Gibson PR Fermentable oligosaccharides, disaccharides, monosaccharides and polyols (FODMAPs) and nonallergic food intolerance: FODMAPs or food chemicals? *Therap Adv Gastroenterol* 2012; 5:261–268. 10.1177/1756283X11436241 PMC3388522 22778791

[74] Gibson PR Varney J Malakar S Food components and irritable bowel syndrome. *Gastroenterology* 2015; 148:1158–1174. 25680668 10.1053/j.gastro.2015.02.005

[75] Pedersen N Vegh Z Burisch J Ehealth monitoring in irritable bowel syndrome patients treated with low fermentable oligo-, di-, mono-saccharides and polyols diet. *World J Gastroenterol* 2014; 20:6680–6684. 24914395 10.3748/wjg.v20.i21.6680 PMC4047359

[76] Rao SS Yu S Fedewa A Systematic review: dietary fibre and FODMAP-restricted diet in the management of constipation and irritable bowel syndrome. *Aliment Pharmacol Ther* 2015; 41:1256–1270. 25903636 10.1111/apt.13167

[77] Staudacher HM Irving PM Lomer MC Mechanisms and efficacy of dietary FODMAP restriction in IBS. *Nat Rev Gastroenterol Hepatol* 2014; 11:256–266. 24445613 10.1038/nrgastro.2013.259

[78] Biesiekierski JR Peters SL Newnham ED No effects of gluten in patients with self-reported non-celiac gluten sensitivity after dietary reduction of fermentable, poorly absorbed, short-chain carbohydrates. *Gastroenterology* 2013; 145:320-8.e1–320-8.e3. 23648697 10.1053/j.gastro.2013.04.051

[79] Murray K Wilkinson-Smith V Hoad C Differential effects of FODMAPs (fermentable oligo-, di-, mono-saccharides and polyols) on small and large intestinal contents in healthy subjects shown by MRI. *Am J Gastroenterol* 2014; 109:110–119. 24247211 10.1038/ajg.2013.386 PMC3887576

[80] Shepherd SJ Gibson PR Fructose malabsorption and symptoms of irritable bowel syndrome: guidelines for effective dietary management. *J Am Diet Assoc* 2006; 106:1631–1639. 17000196 10.1016/j.jada.2006.07.010

[81] Escobar MA Jr Lustig D Pflugeisen BM Fructose intolerance/malabsorption and recurrent abdominal pain in children. *J Pediatr Gastroenterol Nutr* 2014; 58:498–501. 24667867 10.1097/MPG.0000000000000232

